# CRISPR-Cas systems: new players in gene regulation and bacterial physiology

**DOI:** 10.3389/fcimb.2014.00037

**Published:** 2014-04-04

**Authors:** Timothy R. Sampson, David S. Weiss

**Affiliations:** ^1^Department of Microbiology and Immunology, Microbiology and Molecular Genetics Program, Emory University School of MedicineAtlanta, GA, USA; ^2^Emory Vaccine Center, Emory University School of MedicineAtlanta, GA, USA; ^3^Yerkes National Primate Research Center, Emory University School of MedicineAtlanta, GA, USA; ^4^Division of Infectious Diseases, Department of Medicine, Emory University School of MedicineAtlanta, GA, USA

**Keywords:** CRISPR-Cas, Cas9, post-transcriptional regulation of gene expression, bacterial pathogenesis, *Francisella novicida*

## Abstract

CRISPR-Cas systems are bacterial defenses against foreign nucleic acids derived from bacteriophages, plasmids or other sources. These systems are targeted in an RNA-dependent, sequence-specific manner, and are also adaptive, providing protection against previously encountered foreign elements. In addition to their canonical function in defense against foreign nucleic acid, their roles in various aspects of bacterial physiology are now being uncovered. We recently revealed a role for a Cas9-based Type II CRISPR-Cas system in the control of endogenous gene expression, a novel form of prokaryotic gene regulation. Cas9 functions in association with two small RNAs to target and alter the stability of an endogenous transcript encoding a bacterial lipoprotein (BLP). Since BLPs are recognized by the host innate immune protein Toll-like Receptor 2 (TLR2), CRISPR-Cas-mediated repression of BLP expression facilitates evasion of TLR2 by the intracellular bacterial pathogen *Francisella novicida*, and is essential for its virulence. Here we describe the Cas9 regulatory system in detail, as well as data on its role in controlling virulence traits of *Neisseria meningitidis* and *Campylobacter jejuni*. We also discuss potential roles of CRISPR-Cas systems in the response to envelope stress and other aspects of bacterial physiology. Since ~45% of bacteria and ~83% of Archaea encode these machineries, the newly appreciated regulatory functions of CRISPR-Cas systems are likely to play broad roles in controlling the pathogenesis and physiology of diverse prokaryotes.

## Introduction

CRISPR (clustered, regularly interspaced, short, palindromic repeats)—Cas (CRISPR-associated) systems are adaptive, sequence specific, nucleic acid restriction machineries found in many bacteria and Archaea (Makarova et al., 2011). These systems provide prokaryotes with an effective defense against mobile genetic elements, in particular bacteriophages, plasmids, and transposons (Barrangou et al., 2007; Marraffini and Sontheimer, 2008; Garneau et al., 2010; Bikard et al., 2012). The defining feature of CRISPR-Cas systems is a chromosomal array consisting of short, repetitive, and sometimes palindromic sequences, which are interspersed by short, unique, spacer sequences. Such arrays were first identified over 25 years ago, during sequencing of the *iap* gene in *E. coli* (Ishino et al., 1987), and subsequently named CRISPR arrays (which are transcribed into CRISPR RNAs, or crRNA). However, these stretches of repeat sequences had no known function until 2007, when Barrangou et al. demonstrated that they function with associated *cas* genes as adaptive restriction machineries against bacteriophage infection (Barrangou et al., 2007).

**Figure 1:**
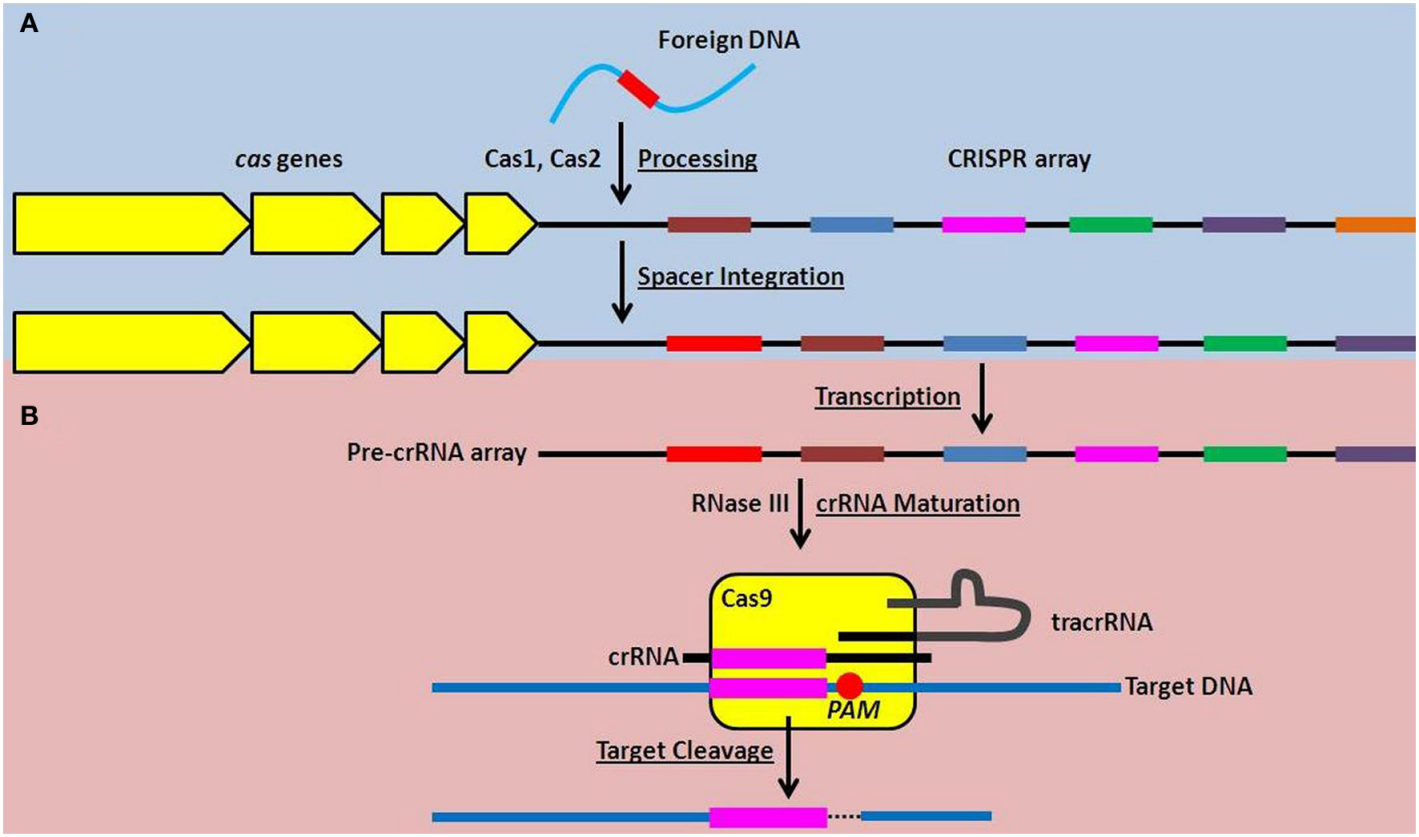
**Function of the Type II CRISPR-Cas system in adaptive nucleic acid restriction. (A)** Foreign DNA is recognized by Cas1 and Cas2 and is processed into a new spacer sequence (red) within the CRISPR array (Adaptation phase, blue). **(B)** To restrict foreign DNA, the CRISPR array is transcribed as a single transcript (pre-crRNA array) and matured into small targeting crRNAs in a process requiring RNase III and tracrRNA. The dsRNA complex of crRNA and tracrRNA is associated with Cas9 and the spacer sequence within the crRNA can hybridize to complementary DNA sequences. Cas9 then mediates cleavage of the targeted DNA downstream of the proto-spacer adjacent motif, or PAM, highlighted by the red circle (Effector phase, pink).

Over the past 5 years, incredible progress has been made in elucidating the molecular mechanisms of action of CRISPR-Cas systems, revealing their roles in a form of RNA-directed nucleic acid interference. Briefly, the entire crRNA array is transcribed as a single, long transcript, that is subsequently processed into individual crRNAs (Figure 1), each containing one spacer sequence and portions of the repeat sequence at both the 5′ and 3′ ends (Golovliov et al., 2003; Brouns et al., 2008; Hale et al., 2009; Pougach et al., 2010; Deltcheva et al., 2011). Cas proteins interact with the processed crRNAs which target the resulting RNA:protein complex to foreign nucleic acids (Figure 1). Specifically, the spacer sequence in each crRNA hybridizes to complementary sequences in nucleic acid targets, ultimately triggering the cleavage of the target by the associated Cas proteins (Figure 1) (Barrangou et al., 2007; Marraffini and Sontheimer, 2008; Garneau et al., 2010; Bikard et al., 2012).

CRISPR-Cas systems are also uniquely adaptive. In a currently incompletely defined process, it is thought that the Cas proteins Cas1 and Cas2 recognize foreign nucleic acid that has entered the prokaryotic cell, and process it into a new spacer sequence(s) that is then integrated into the crRNA array (Figure 1) (Datsenko et al., 2012; Fineran and Charpentier, 2012). This allows the individual bacterial cell and its progeny to subsequently target the foreign nucleic acid if encountered again (Barrangou et al., 2007; Datsenko et al., 2012; Yosef et al., 2012). This provides prokaryotes encoding CRISPR-Cas systems with an unprecedented adaptive mechanism to prepare for, and mitigate, future threats.

Interestingly, some bacteria encode spacers within the crRNA array that have sequence identity to chromosomal loci. In many such cases, the associated *cas* genes have degenerated, providing an explanation for why the chromosome itself is not targeted (Stern et al., 2010). However, in some bacteria, this does not appear to be the case, and the associated *cas* genes are still intact. Due to their functionality in sequence-specifically targeting and cleaving nucleic acids, this presence of “self-targeting” crRNAs has led to the hypothesis that CRISPR-Cas systems may have an additional functionality as regulatory elements.

## The type II CRISPR-Cas system as a post-transcriptional regulator

While all known CRISPR-Cas systems contain Cas1 and Cas2, three different types (Type I, II, and III) are each characterized by unique Cas proteins involved in maturation of the crRNAs, targeting of foreign nucleic acid, and nucleic acid cleavage (Makarova et al., 2011). The Type II CRISPR-Cas system is defined by the presence of a large (~1000–1600 amino acids) endonuclease, Cas9, whose structure has recently been solved (Deltcheva et al., 2011; Chylinski et al., 2013; Fonfara et al., 2013; Jinek et al., 2014). Predominantly, Type II systems are encoded in the genomes of pathogenic (including *Neisseria meningitidis*, *Campylobacter jejuni*, *Legionella pneumophila*, *Listeria monocytogenes*, and *Francisella novicida* Makarova et al., 2011; Sampson et al., 2013) and commensal bacteria that interact with eukaryotic hosts. Type II CRISPR-Cas systems are further characterized by the requirement for a unique, accessory RNA, the *trans*-activating CRISPR RNA (tracrRNA), as well as RNase III, for maturation of crRNAs (Figure 1) (Deltcheva et al., 2011; Jinek et al., 2012; Chylinski et al., 2013). Cas9 is involved as a scaffold for maturation of crRNAs, and is required for cleavage of the double-stranded DNA target (Figure 1) (Deltcheva et al., 2011; Gasiunas et al., 2012; Jinek et al., 2012).

**Figure 2:**
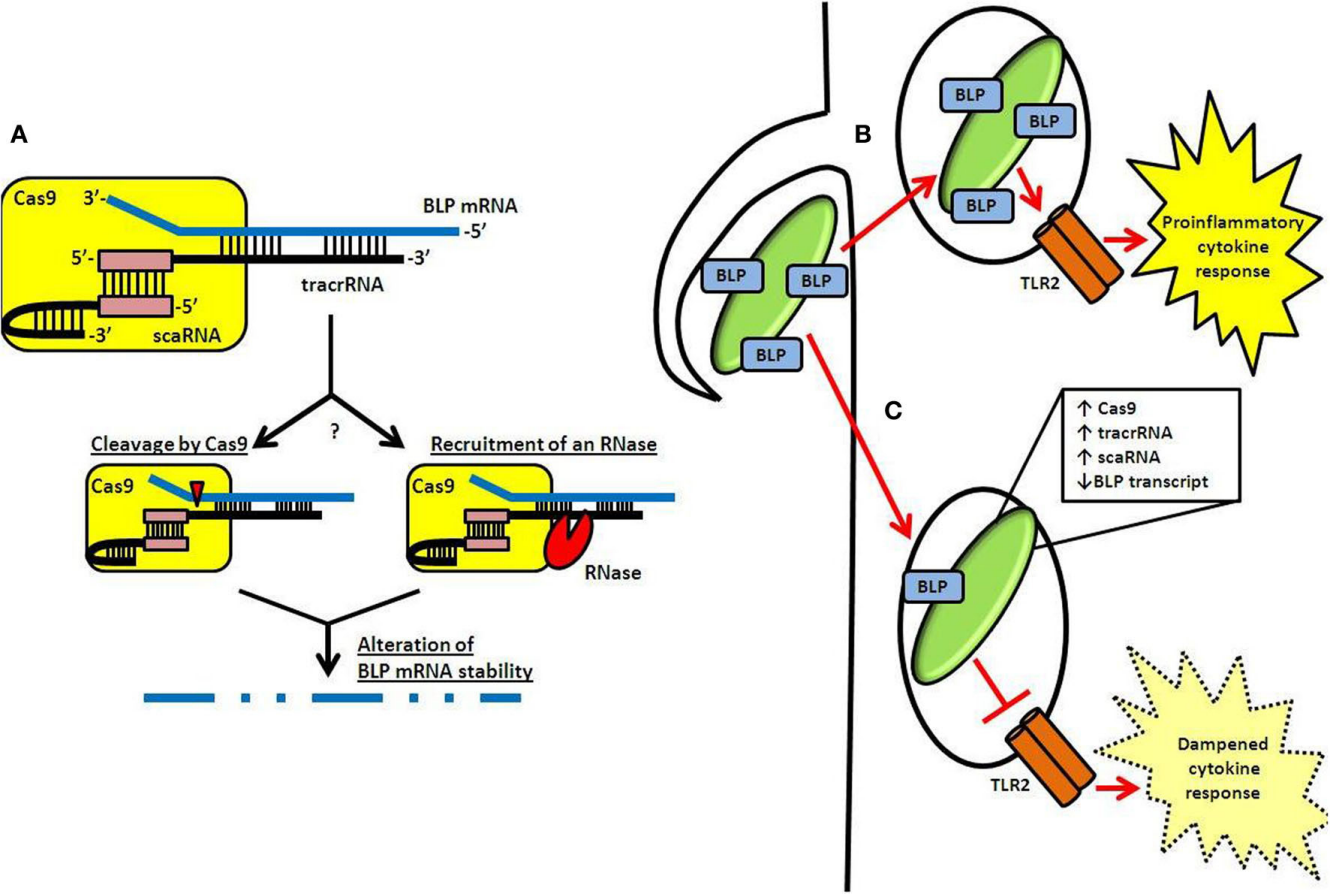
**CRISPR-Cas-mediated gene regulation and innate immune evasion by *F. novicida*. (A)** A dual-RNA complex consisting of the tracrRNA and scaRNA forms through interaction of a sequence identical to the CRISPR repeat (pink box). This dsRNA structure is associated with *F. novicida* Cas9 and allows the free portion of the tracrRNA to interact through a non-identity interaction with mRNA encoding the BLP (*FTN_1103*; blue). Subsequently, the stability of the BLP mRNA is altered, possibly via catalytic activity of Cas9 (cleavage event indicated by red triangle) or by an unknown RNase (red sector). **(B)** Following entry into host cells, TLR2 is capable of detecting BLPs present in the *F. novicida* envelope. This leads to activation of a proinflammatory cytokine response. **(C)** However, by upregulating CRISPR-Cas components after entry and while in the phagosome, *F. novicida* limits its BLP content and dampens the activation of TLR2, leading to decreased proinflammatory cytokine signaling.

Recently, we demonstrated that specific components of the Type II CRISPR-Cas system in the Gram-negative intracellular pathogen *Francisella novicida* (one of two CRISPR-Cas systems present in this species Schunder et al., 2013) regulate the expression of an endogenous transcript encoding a bacterial lipoprotein (BLP) (Jones et al., 2012b; Sampson et al., 2013). Cas9, together with tracrRNA as well as a small RNA currently unique to the *F. novicida* system, termed scaRNA (small, CRISPR-Cas-associated RNA), form a dual RNA:protein complex capable of targeting the BLP transcript (Figure 2A) (Sampson et al., 2013). Interestingly, in contrast to its accessory role in canonical DNA targeting by Cas9, tracrRNA displays significant sequence complementarity to the BLP mRNA, and is thought to function in a targeting role (Figure 2A) (Sampson et al., 2013).

scaRNA is predicted to hybridize to the tracrRNA at a sequence identical to the CRISPR repeat, forming a dsRNA structure that may interact with Cas9 in a similar fashion as the crRNA:tracrRNA complex within the canonical DNA-targeting CRISPR-Cas system (Figure 2A) (Deltcheva et al., 2011; Chylinski et al., 2013; Sampson et al., 2013). While the exact function of the scaRNA is unknown, the current hypothesis is that it serves to stabilize the tracrRNA in such a way that tracrRNA can subsequently interact with the BLP transcript. This could be through direct changes in the structure of the RNA after hybridization, or by altering the way in which the RNAs interact with Cas9. Further structural and stoichiometric studies will help to elucidate how these RNAs interact with Cas9 to target the BLP mRNA.

Targeting by the Cas9:tracrRNA:scaRNA machinery results in drastically lowered levels of BLP mRNA, through a process that alters the stability of the transcript (Sampson et al., 2013). Exactly how the stability of the BLP mRNA is altered is unknown. Surprisingly, this regulation does not require the amino acid residues essential to the endonuclease activity of Cas9 proteins (Jinek et al., 2012; Fonfara et al., 2013; Sampson et al., 2013). This may suggest that Cas9 has redundant endonuclease motifs, each capable of acting on the targeted BLP transcript. Alternatively, an accessory RNase may be involved (Figure 2A). While a number of different RNases were tested for their function in regulation of this BLP, no single RNase mutant had an apparent regulatory defect. One specific RNase, RNase E, could not be analyzed using this approach since it is an essential gene (Heidrich and Vogel, 2013a). Other studies have shown that RNase E can be involved in the modulation of mRNA stability, and it is therefore possible that it may also act as an accessory for CRISPR-Cas-mediated regulation (Heidrich and Vogel, 2013a).

The predicted requirements for targeting the Cas9:tracrRNA:scaRNA system to the BLP transcript also differ considerably with those for targeting of foreign DNA by the canonical CRISPR-Cas system. Targeting of the Cas9 endonuclease to foreign DNA requires a crRNA with near 100% sequence identity to the target (Makarova et al., 2011). Surprisingly, crRNAs are not required for targeting of BLP mRNA (Sampson et al., 2013), and there does not appear to be a spacer sequence within the crRNA array that has sequence complementarity to this transcript, making this regulatory process distinct from the canonical DNA targeting action of Cas9. Instead, the BLP mRNA is targeted by the tracrRNA, which only displays partial sequence complementarity to the transcript. This may suggest that only small stretches of sequence complementarity, or seed sequences, are important for initiating and establishing interaction of the tracrRNA and BLP mRNA, as has been demonstrated for nucleic acid targeting by other CRISPR-Cas systems (Semenova et al., 2011; Kunne et al., 2014).

An interesting question is why BLP mRNA is apparently targeted, yet the BLP gene encoded in the chromosome is not targeted for disruption. It has been observed that targeting of the bacterial chromosome results in loss of either that chromosomal sequence or the CRISPR-Cas system itself (Stern et al., 2010; Dy et al., 2013; Vercoe et al., 2013). Since this is not the case in *F. novicida*, it strongly suggests that the DNA is not targeted for cleavage. The imperfect complementarity between the tracrRNA and the BLP mRNA would not be predicted to mediate targeting of the chromosomal DNA, based on canonical CRISPR-Cas targeting. Therefore, this lack of 100% complementarity could be an important safeguard to effectively prevent DNA targeting while nonetheless promoting targeting of mRNA. Understanding the structural and sequence requirements of these interactions will be important for elucidating how prokaryotes control the different activities of CRISPR-Cas systems.

While the data discussed support a model whereby *F. novicida* Cas9 post-transcriptionally modulates the stability of the BLP transcript, other potential models exist as well. It is theoretically possible that *F. novicida* Cas9 binds DNA but does not cleave it, and thereby physically blocks transcription. There is precedence for this, at least in synthetically mutated Cas9 proteins (dCas9), which lack endonuclease activity, but retain the ability to bind DNA (Bikard et al., 2013; Cheng et al., 2013; Esvelt et al., 2013; Mali et al., 2013; Perez-Pinera et al., 2013; Qi et al., 2013). Upon being guided to a target gene or its promoter, dCas9 can effectively prevent transcription by blocking access of RNA polymerase (RNAP) to the promoter and/or preventing RNAP elongation. This system has been successfully utilized as a tool in order to repress target genes in numerous biological systems (Bikard et al., 2013; Cheng et al., 2013; Esvelt et al., 2013; Mali et al., 2013; Perez-Pinera et al., 2013; Qi et al., 2013). A lack of *F. novicida* Cas9 endonuclease activity would be consistent with the observation that none of the conserved residues within any of the predicted endonuclease motifs of the protein were necessary for repression of the BLP transcript (Sampson et al., 2013). However, recent data demonstrates that, in fact, *F. novicida* Cas9 is fully capable of targeting and cleaving DNA substrates, at least *in vitro* (Fonfara et al., 2013). The finding that *F. novicida* Cas9 can cleave DNA, yet the BLP gene is nonetheless present in the genome and the protein is produced (Jones et al., 2012b; Fonfara et al., 2013; Sampson and Weiss, 2013; Sampson et al., 2013), supports the hypothesis that the DNA is not targeted while BLP mRNA is. Furthermore, this alternative transcription inhibition model would not explain the observed changes in the BLP transcript's stability after treatment of cells with rifampin which blocks transcription, nor the presence of the BLP transcript in association with Cas9 (Sampson et al., 2013).

Assuming that *F. novicida* Cas9 does target RNA, it is possible that this is not a feature common to all Cas9 proteins. While similar to other Cas9 proteins, *F. novicida* Cas9 does have regions of significant sequence dissimilarity which may alter its function in targeting DNA or RNA (Makarova et al., 2011; Chylinski et al., 2013; Fonfara et al., 2013; Sampson et al., 2013). There is precedence for such a scenario in Type III CRISPR-Cas systems. The Type III-A and III-B systems have slight differences in the structures of their targeting complexes (Cascade) which may account for their differential ability to target DNA substrates (III-A) or RNA substrates (III-B) (Heidrich and Vogel, 2013b; Rouillon et al., 2013; Spilman et al., 2013; Staals et al., 2013). Continued dissection of the molecular mechanism of Cas9 function will provide answers to these and other critical remaining questions, in particular focusing on structure and function comparisons between Cas9 variants (Fonfara et al., 2013; Jinek et al., 2014; Sternberg et al., 2014).

## Role of CRISPR-Cas-mediated gene regulation in pathogenesis

The bacterial pathogen *Francisella novicida*, a relatively rare cause of disease in humans, evades detection by the host innate immune system and replicates within host cells (Jones et al., 2012a). *F. novicida* has numerous mechanisms by which to subvert the function of host macrophages as well as other cells. Once taken up by macrophages, this pathogen enters the phagosome, a compartment containing numerous antimicrobials as well as innate immune recognition receptors (Jones et al., 2012a). One such receptor is Toll-like Receptor 2 (TLR2), which detects BLPs (Aliprantis et al., 1999; Brightbill et al., 1999). Activation of TLR2 results in a pro-inflammatory response which recruits and activates immune cells, and acts to combat and clear the bacterial pathogen.

Utilizing Cas9, tracrRNA, and scaRNA as regulators, *F. novicida* represses the expression of the targeted BLP, significantly lowering overall BLP levels in its envelope by roughly 2-fold (Figure 2C) (Jones et al., 2012b; Sampson et al., 2013). This allows the pathogen to effectively dampen TLR2 activation, facilitating its survival within the host. In the absence of this CRISPR-Cas-mediated regulation, *F. novicida* elicits a significant TLR2-dependent inflammatory response (Figure 2B), as revealed by the fact that *cas9*, tracrRNA, and scaRNA deletion mutants induce a much greater inflammatory response than wild-type bacteria (Sampson et al., 2013). Not only is this inflammatory response dependent on TLR2, but it is also dependent on the over-expression of BLP, as strains lacking both the regulatory components and the BLP elicit a response that is limited to near wild-type levels (Jones et al., 2012b; Sampson et al., 2013). Furthermore, deletion mutants lacking these CRISPR-Cas components are highly attenuated (over 1000 fold) (Sampson et al., 2013). In addition, *cas9*, tracrRNA, and scaRNA deletion mutants are unable to induce a lethal infection of mice, further emphasizing their importance as regulators of virulence in *F. novicida* (Sampson et al., 2013).

While the *F. novicida* CRISPR-Cas system is currently the only known example of a Cas9 system acting naturally in a regulatory capacity, there have been observations of other species utilizing Cas9 as a virulence factor. In a human lung epithelial cell model, Cas9 is essential for attachment of *Neisseria meningitidis* to the host cell surface, as well as both invasion and intracellular replication (Sampson et al., 2013). Additionally, Cas9 is essential for attachment and invasion of *Campylobacter jejuni* in a colorectal epithelial cell model (Louwen et al., 2013). The precise mechanism by which Cas9 functions as a virulence factor in these organisms is not yet known. However, based on the established role of Cas9 as a regulator of gene expression in *F. novicida*, it is likely that Cas9 acts in combination with tracrRNA or an alternative, unidentified small RNA, to regulate the levels of specific transcripts, ultimately leading to the control of virulence properties.

Additionally, the role of Cas9 as a *Campylobacter* virulence factor correlated with specific strains encoding the Cst-II sialyltransferase, and which produce a sialylated lipooligosaccharide (Louwen et al., 2013). It is interesting to hypothesize that CRISPR-Cas-mediated regulation may act not only to allow *C. jejuni* to efficiently attach to host cells, but also to mask its surface from detection by host receptors, and prevent activation of host defenses, such as the complement system. Since the known regulatory target of Cas9 in *Francisella* is a membrane BLP, and these additional examples of a contribution of Cas9 to virulence traits involve attachment of the bacterial cell to the host cell surface, it is interesting to speculate that CRISPR-Cas systems may generally act as regulators of envelope composition and structure.

## Role of CRISPR-Cas systems in the response to envelope stress

The regulation of the bacterial envelope is especially important during times of membrane stress, in order to resist and combat this stress, and to promote bacterial survival. Interestingly, it has been observed that expression of CRISPR-Cas components in several bacterial species can be induced following envelope stress. For instance, in *E. coli* when a membrane-targeted GFP is overexpressed, the downstream envelope stress response triggers the upregulation of CRISPR-Cas system expression (Perez-Rodriguez et al., 2011). Additionally, CRISPR-Cas systems in other bacterial and archaeal species, including *Streptococcus thermophilus* and *Sulfolobus islandicus*, have been shown to be induced in the presence of bacteriophage (Young et al., 2012; Quax et al., 2013), suggesting that the envelope stress which occurs during attachment and entry of bacteriophage may be a signal to activate CRISPR-Cas systems. Envelope stress may further serve as a signal of increased cell permeability, a condition that would likely increase the chance of foreign nucleic acid uptake. Thus, induction of CRISPR-Cas systems at times of envelope stress might act to prepare the cell for incoming foreign nucleic acid and prevent acquisition of harmful genetic elements. In addition, since CRISPR-Cas systems have been shown to regulate envelope components, and these systems are induced in response to membrane stress, it is tempting to speculate that the regulatory roles of these machineries may also serve to combat this stress. For example, the regulation of membrane BLP composition, as observed in *F. novicida* (Jones et al., 2012b; Sampson et al., 2013), in addition to promoting evasion of the host innate immune response, may act to alter or enhance the integrity of the bacterial envelope.

## Role of other CRISPR-Cas components and systems in bacterial physiology

In addition to Cas9's role as a virulence factor in the bacterial pathogens *F. novicida, N. meningitidis*, and *C. jejuni*, CRISPR-Cas systems in other bacteria have been identified as having potential roles in virulence as well. Cas2, present within a Type II CRISPR-Cas system, is important for the ability of *Legionella pneumophila* to replicate within amoebae (Gunderson and Cianciotto, 2013). Since amoebae are thought to be important for *L. pneumophila* survival in the environment (Rowbotham, 1986; Fields, 1996; Abu Kwaik et al., 1998), the role of Cas2 in intracellular amoebic survival may play a role in its survival in the environment. In addition, in strains encoding the Type II system, it may even promote subsequent transmission to human hosts (Gunderson and Cianciotto, 2013). Exactly how Cas2 functions to mediate *Legionella* intracellular survival in amoebae is unknown. It is hypothesized to have an alternative function in conjunction with currently unidentified small RNAs, either in their processing or in the alteration of mRNA stability (Gunderson and Cianciotto, 2013). Interestingly, Cas9 has no observed role in *L. pneumophila* survival in amoebae (Gunderson and Cianciotto, 2013). Conversely, Cas2 has no observed role in the ability of *F. novicida* to modulate BLP expression, nor intracellular survival or virulence (Sampson et al., 2013), demonstrating that while Type II CRISPR-Cas systems have similar genetic architectures, different species may have co-opted alternative components for functions distinct from defense against foreign nucleic acids.

Type I CRISPR-Cas systems have also been implicated in aspects of bacterial physiology beyond their now canonical function in foreign nucleic acid defense. The Type I CRISPR-Cas system in *Pseudomonas aeruginosa* has been shown to play a role in modulating the production of biofilms (Zegans et al., 2009; Cady and O'Toole, 2011). While the exact regulatory mechanism has not been elucidated, the data suggest that the CRISPR-Cas system interacts with a specific gene within a chromosomally integrated prophage, inhibiting biofilm formation (Zegans et al., 2009; Cady and O'Toole, 2011). It is unclear if the CRISPR-Cas system targets the chromosomal DNA or the prophage transcript, but it is known that this regulation requires the Cas proteins involved in crRNA maturation, as well as those involved in targeting/degradation. Further, this regulatory activity depends on a specific crRNA with sequence identity to the prophage gene (Zegans et al., 2009; Cady and O'Toole, 2011). Interestingly, this crRNA does not exhibit 100% complementarity to its regulatory target. Similar to *F. novicida* Cas9 targeting of BLP mRNA, this is a non-identity interaction, perhaps providing a reason for why chromosomal targeting by the *Pseudomonas* CRISPR-Cas system would not result in a lethal event. Given that biofilm formation is a critical aspect of the pathogenic life cycle of *P. aeruginosa* (Gellatly and Hancock, 2013), it is likely that this example of CRISPR-Cas-mediated regulation plays a vital role in infection.

Another Type I CRISPR-Cas system with regulatory attributes is found in the soil bacterium *Myxococcus xanthus*. Three genes, *devT*, *devR*, and *devS*, corresponding to *cas8*, *cas7*, and *cas5* respectively, have been shown to be necessary for sporulation and fruiting body development (Thony-Meyer and Kaiser, 1993; Boysen et al., 2002; Viswanathan et al., 2007). Specifically, it was observed that *devT* (*cas8*) mutants had significant delays in aggregation, sporulation, and chemotaxis. This correlated with low levels of transcript for a necessary activator of fruiting body formation (Boysen et al., 2002). It is not known if the crRNA array is necessary for fruiting body formation, however it does encode two spacers with complementarity to chromosomal loci, one of which could hybridize to an integrase of a *Myxococcus* bacteriophage and the other that could hybridize to a *cas* gene in an exogenous CRISPR-Cas locus (Viswanathan et al., 2007). How these CRISPR-Cas components interact to ultimately perform this regulatory function remains to be elucidated.

Additionally, the Archaeal Type I system encoded by *Pelobacter* sp. has been demonstrated to play a role in the regulation of gene expression as well (Aklujkar and Lovley, 2010). These species contain a spacer within the crRNA array with sequence identity to the gene encoding a histidyl-tRNA (Aklujkar and Lovley, 2010). Upon expression of this self-targeting spacer within a species related to *Pelobacter* encoding a similar CRISPR-Cas system (but lacking the self-targeting spacer), it was observed that histidyl-tRNA transcript levels were reduced, and that the bacteria exhibited a growth defect (as expected if protein synthesis is slowed by lower levels of a critical tRNA) (Aklujkar and Lovley, 2010). The precise mechanism and how the *cas* genes are involved in this process is yet unknown. In addition to these examples of self-targeting spacers involved in gene regulation through unknown mechanisms, there are numerous examples of self-targeting crRNA spacers (Stern et al., 2010). However, it is unclear if they are indeed involved in regulation. For example, *Aggregatibacter actinomycetemcomitans* encodes a spacer putatively targeting the important metabolic enzyme *glgP* (Jorth and Whiteley, 2012). While this crRNA is transcribed and processed, it is not known if it acts as a regulator of *glgP* production (Jorth and Whiteley, 2012). Future study of such spacers will likely reveal a plethora of regulatory functions for CRISPR-Cas systems in diverse bacteria.

There also exist examples in which crRNAs may have regulatory roles, even in the absence of Cas proteins. *Listeria monocytogenes* encodes an isolated crRNA locus, consisting of five identical repeats, and four unique spacer sequences (Mandin et al., 2007; Sesto et al., 2014). This locus, termed rliB, is not adjacent to any known *cas* genes, and is present even within *L. monocytogenes* strains that are devoid of any *cas* genes (Mandin et al., 2007; Sesto et al., 2014). rliB is processed by the bifunctional polynucleotide phosphorylase (PNPase), which has exoribonuclease activity (Sesto et al., 2014). The rliB crRNA has significant sequence complementarity to the transcripts for a two component system, a transcriptional regulator, and the *feoAB* iron transport system (Mandin et al., 2007). In fact, rliB is capable of hybridizing to and repressing production of these transcripts (Mandin et al., 2007). Since *feoAB* is an important virulence factor in numerous organisms, it is likely that rliB plays an important role in the virulence of *L. monocytogenes*. Interestingly, this orphaned system is still capable of acting canonically against plasmid transformation, provided there are *cas* genes produced in an exogenous locus. However it is unknown if this occurs through targeting of DNA or RNA substrates (Sesto et al., 2014).

While the aforementioned examples of alternative CRISPR-Cas function focus on regulatory roles, there may be more indirect mechanisms by which CRISPR-Cas systems contribute to virulence. For example, it has been observed that both Cas1 and the crRNA array in the Type I CRISPR-Cas system of *E. coli* play a role in DNA repair (Babu et al., 2011). Given that Cas1 is present in all known CRISPR-Cas systems, it is interesting to think that this gene may have a broad function in DNA repair in other species as well. Furthermore, bacterial DNA damage is thought to occur due to the action of specific host defenses during infection, in particular the production of radical nitrogen and oxygen species (Suvarnapunya et al., 2003). It is therefore interesting to consider that Cas1 may be able to provide bacterial pathogens some redundancy in their capability to repair DNA damage incurred during infection.

## Conclusions

While now very well established to play roles in bacteriophage and foreign genetic element defense, the alternative functions that CRISPR-Cas systems play in the ability of bacterial pathogens to evade and dampen host defenses, and ultimately survive and replicate within the host, have only recently begun to be appreciated. Furthermore, with the continued observations that some CRISPR-Cas systems can target RNA substrates (Hale et al., 2009; Spilman et al., 2013; Terns and Terns, 2013), this raises the strong possibility that regulation of endogenous genes by CRISPR-Cas systems can occur without the negative consequences of targeting the bacterial chromosome (Stern et al., 2010; Dy et al., 2013; Vercoe et al., 2013). Given that CRISPR-Cas systems are encoded in the genomes of numerous prokaryotes (including ~45% of bacteria and ~83% of Archaea) (CRISPRdb, 23 Jan 2014) (Grissa et al., 2007), it is likely that numerous examples of alternative functions in gene regulation, virulence and physiology will be uncovered in the future.

### Conflict of interest statement

The authors declare that the research was conducted in the absence of any commercial or financial relationships that could be construed as a potential conflict of interest.
